# Sex-specific contribution of lipid accumulation product and cardiometabolic index in the identification of nonalcoholic fatty liver disease among Chinese adults

**DOI:** 10.1186/s12944-021-01617-3

**Published:** 2022-01-13

**Authors:** Yiting Liu, Wei Wang

**Affiliations:** Department of Physical Examination Center, The First Affiliated Hospital, China Medical University, Shenyang, China

**Keywords:** Nonalcoholic fatty liver disease, Lipid accumulation product, Cardiometabolic index, Sex

## Abstract

**Background:**

Lipid accumulation product (LAP) and cardiometabolic index (CMI) are two novel obesity-related indexes associated with enhancing metabolic disease (MD) risk. Current evidences suggest that the differences in sex hormones and regional fat distribution in both sexes are directly correlated with MD and nonalcoholic fatty liver disease (NAFLD) risk. Hence, NAFLD incidences reflect sex differences. Herein, we examined the accuracy of LAP and CMI in diagnosing NAFLD in both sexes.

**Methods:**

Overall, 14,407 subjects, who underwent health check-up in the northeastern China, were enrolled in this study, and their corresponding LAP and CMI were calculated. Abdominal ultrasonography was employed for NAFLD diagnosis. Multivariate analyses were analyzed potential correlations between LAP and/or CMI and NAFLD. Odds ratios (ORs) and 95% confidence intervals (CIs) were evaluated. Receiver operating characteristic curve analyses was executed for the exploration of the diagnostic accuracies. Areas under the curves (AUCs) with 95%CIs were calculated.

**Results:**

NAFLD prevalence increased with elevated quartiles of LAP and CMI in both sexes. In multivariate logistic regression analyses, LAP and CM expressed as continuous variables or quartiles, significantly correlated with NAFLD. The ORs for the top versus bottom quartile of LAP and CMI for NAFLD were 13.183 (95%CI = 8.512–20.417) and 8.662 (95%CI = 6.371–11.778) in women and 7.544 (95%CI = 5.748–9.902) and 5.400 (95%CI = 4.297–6.786) in men. LAP and CMI exhibited larger AUCs, compared to other obesity-related indexes in terms of discriminating NAFLD. The AUCs of LAP and CMI were 0.860 (95%CI = 0.852–0.867) and 0.833 (95%CI = 0.825–0.842) in women and 0.816 (95%CI = 0.806–0.825) and 0.779 (95%CI = 0.769–0.789) in men.

**Conclusions:**

LAP and CMI are convenient indexes for the screening and quantification of NAFLD within a Chinese adult population. Their associations with NAFLD are substantially greater in women than men.

## Background

With the recent advancements in social economy and subsequent alterations in dietary structure and living habits, nonalcoholic fatty liver disease (NAFLD) prevalence has gradually become a principal community health challenge throughout the world [1]. NAFLD represents an extensive range of liver diseases, for instance, nonalcoholic steatosis, nonalcoholic steatohepatitis, liver cirrhosis and hepatocellular carcinoma [2]. It is clearly associated with obesity, type 2 diabetes mellitus, dyslipidemia, metabolic syndrome (MS) and cardiovascular disease [3]. Thus, early NAFLD identification and diagnosis in a simple and effective manner is essential to prevent and delay the advancement of NAFLD and occurrence of its related complications.

Lipid accumulation product (LAP), which is independently calculated for males and females, is an alternative index for excess lipid accumulation [4]. Several studies revealed that LAP is a strong anthropometric indicator to predict diabetes, MS, insulin resistance and NAFLD [5–8]. Current evidence suggests that the differences in sex hormones and regional fat distribution in both sexes are directly correlated with regulating metabolic disorder (MD) and NAFLD. Hence, NAFLD prevalence reflects sex differences [9]. However, very few studies examined how sex differences affected the relationship between LAP and NAFLD.

Recently, cardiometabolic index (CMI) was proposed as a simple and reliable surrogate indicator for diabetes recognition [10]. A community-based study involving 11,478 participants from rural Northeastern China estimated diabetes prevalence using CMI, and supported CMI as an economic, stable and dose-dependent index for screening and discriminating diabetes among a general Chinese population [11]. Several studies also revealed its value in identifying the deterioration of metabolic profile and cardiovascular diseases, including hypertension, hyperuricemia, arterial stiffness, ischemic stroke, and left ventricular geometry abnormality [12–16]. Considering that MS is closely associated with NAFLD, there may be an association between CMI and NAFLD. Additionally, CMI and NAFLD prevalence often vary by sex, so there may potentially be a sex -specific difference in CMI action.

Herein, a cross-sectional investigation was conducted to explore the clinical role of LAP and CMI stratified by sex-specific quartiles in the prevalence of NAFLD, and to present a theoretical foundation for the screening of NAFLD among a Chinese adult population.

## Methods

### Study population

A cross-sectional epidemiological investigation was performed in subjects (age ≥ 18 years) who underwent their physical examinations at the First Affiliated Hospital of the China Medical University between January 2019 and December 2019. The following subjects were excluded from analysis: (1) subjects who had long-term excessive drinking habit (alcohol intake exceeded 20 g per day for male or 10 g per day for female) [17]; (2) subjects who had viral hepatitis, drug-linked liver injury, autoimmune liver disease and other specific illnesses (i.e. Reye’s syndrome, acute fatty liver of pregnancy, Wilson’s disease) that can lead to fatty liver; (3) subjects who had consumed hepatoprotective drugs; (4) subjects with severe liver and kidney dysfunction; (5) missing data. The study was approved by the Ethics Committee of China Medical University (approval number: 2019–77). The informed consent requirement was exempted owing to the retrospective nature of this research.

### Data collection

Demographic characteristics and general information of the subjects were collected by self-administered questionnaire regarding age, sex, medical history, family history, medication history and alcohol consumption. Standard weight and height were assessed while subjects had on light clothing and no shoes. Waist circumference (WC) was determined, employing a soft tape, at the midpoint of the distance between the lower edge of the costal ridge and the upper border of the iliac crest. Systolic blood pressure (SBP) and diastolic blood pressure (DBP) were measured twice following a 5 min rest, using an electronic sphygmomanometer. The average of the two values was documented as the final blood pressure. Upon overnight fasting, the samples of venous blood were accumulated and measured for biochemical markers, including serum triglyceride (TG), total cholesterol (TC), high-density lipoprotein cholesterol (HDL-C), low density lipoprotein cholesterol (LDL-C), fasting blood glucose (FPG), serum uric acid (UA), serum creatinine (Scr), blood urea nitrogen (BUN), white blood cell count (WBC), alanine aminotransferase (ALT), aspartate aminotransferase (AST) and γ-glutamyltransferase (γ-GGT) using a Cobas 8000 automatic biochemical analyzer.

### Definitions

Following the Asia-Pacific Working Party criteria, the NAFLD diagnosis was made according to the results of abdominal ultrasonography scans, supporting the presence of fatty liver disease. This excluded cases of excessive alcohol consumption, hepatitis virus, hepatotoxic medicines and autoimmune liver diseases, based on the results of self-administered questionnaire [17]. Fatty liver was evaluated according to the presence or absence of hepatic steatosis, based on echo patterns, namely, hepatic versus nephritic diffuse hyper-echogenicity, limited visualization of intrahepatic structures, and ultrasound beam attenuation without semi-quantitative indices. All ultrasonographic investigations were conducted by a trained and experienced professional radiologist using a 3.5-MHz ultrasonic probe (Acuson X300, Siemens, Germany).

The body mass index (BMI) was evaluated as weight in kilograms divided by the square of height in meters. Waist-to-height (WHtR) was defined as WC divided by height in centimeters. LAP was ascertained employing the formula given below [4]: LAP = TG (mmol/L) × [WC (cm)-58] for women and LAP = TG (mmol/L) × [WC (cm)-65] for men. CMI was assessed with the following equation^9^: CMI = TG (mmol/L)/HDL-C (mmol/L) × WHtR.

Current smoking was described as regular cigarette smoking for over 6 months at the time of physical examination [18]. Regular exercising was described as 30 min of moderate-intensity activity for over three times a week [19].

### Statistical analyses

All analyses were separated by sex. The Kolmogorov-Smirnov assessment was employed to investigate the normal distribution of continuous variables. The normally distributed outcomes were given as mean ± standard deviation (SD), and the intergroup comparisons were fulfilled with the Student’s t test. Non-normally distributed data were given as median with interquartile range, and the intergroup differences were carried out assessment via the Mann-Whitney U test. Categorical outcomes are presented as counts and percentages, and the intergroup differences were assessed via chi-squared test. The LAP and CMI quartiles were divided into four groups: quartile 1 (Q1) (≤P_25_), quartile 2 (Q2) (P_25,_ P_50_), quartile 3 (Q3) (P_50,_ P_75_), and quartile 4 (Q4) (>P_75_). The independent association of LAP and CMI was explored as continuous variables or quartiles with NAFLD occurrence. Upon adjusting for possible confounding variables, a multivariable model was utilized to evaluate the influence of LAP and CMI on NAFLD prevalence. The odds ratios (ORs) and 95% confidence intervals (*CIs*) were presented to predict the effect. The sex -specific estimation of the OR for 1 SD increment in LAP and CMI was obtained to ascertain NAFLD risk. A receiver operator characteristic (ROC) curve assessment was conducted for ascertaining the ability of indicators to predict NAFLD diagnosis, and to confirm the optimal cut-off values. SPSS version 23.0 (IBM, Corp., N.Y., USA) and Stata Software version 16.0 (Stata, Corp., N.Y., USA) were employed for all statistical assessments. Two-tailed *P* values <0.05 was set as statistically significant.

## Results

### Baseline characteristics

Overall, 14,407 eligible subjects (7630 females and 6777 males) were evaluated, with the median age (interquartile range) of 47 (35, 57) years. There were 2030 female participants and 4263 male participants diagnosed with NAFLD, according to the entry criteria, with a prevalence of 26.61 and 62.90%, respectively. The baseline features of the subjects, in terms of their NAFLD status, were separately described in Table 1 for each sex. Overall, NAFLD patients were advanced in age, compared to non-NAFLD subjects, and NAFLD patients exercised less regularly than non-NAFLD participants. Furthermore, regardless of sex, NAFLD patients exhibited significantly elevated BMI, WC, WHtR, LAP, CMI, WBC, SBP, DBP, TG, TC, LDL-C, FPG, ALT, AST, GGT, and UA and reduced HDL-C, relative to non-NAFLD participants. In terms of females, NAFLD patients had a significantly higher BUN than non-NAFLD participants. In terms of males, NAFLD patients had a significantly higher proportion of current smoking than non-NAFLD participants.

**Table 1:** Baseline characteristics of subjects stratified by gender

Variables	Women (*n* = 7630)		χ^2^/Z	*P* value ^a^	Men (*n* = 6777)		χ^2^/Z	*P* value ^a^
	non-NAFLD (*n* = 5600)	NAFLD (*n* = 2030)			non-NAFLD (*n* = 2514)	NAFLD (*n* = 4263)		
Age (years)	41 (32,53)	55 (45,63)	− 28.372	<0.001	47 (35,60)	48 (37,57)	−2.539	0.011
Current smoking (%)	350 (6.25)	112 (6.40)	0.060	0.807	1055 (41.96)	2260 (53.01)	77.264	<0.001
Regular exercising (%)	2948 (52.64)	849 (41.82)	69.775	<0.001	1236 (49.16)	1361 (31.93)	198.831	<0.001
BMI (Kg/m^2^)	22.39 (20.67,24.28)	26.15 (24.29,28.35)	−44.559	<0.001	23.97 (22.19,25.74)	26.90 (25.11,29.00)	−39.727	<0.001
WC (cm)	71 (67,77)	81 (76,86)	−42.645	<0.001	80 (76,85.25)	88 (83,93)	−37.476	<0.001
WHtR	0.44 (0.42,0.48)	0.51 (0.48,0.55)	− 43.521	<0.001	0.47 (0.44,0.50)	0.51 (0.48,0.54)	−36.3	<0.001
LAP (cm.mol/L)	11.00 (6.00,19.56)	35.41 (22.46,54.73)	−48.078	<0.001	16.08 (9.00,25.76)	39.60 (24.13,63.50)	−43.466	<0.001
CMI	0.24 (0.15,0.39)	0.61 (0.39,0.95)	−44.588	<0.001	0.38 (0.25,0.60)	0.81 (0.51,1.31)	−38.499	<0.001
WBC (10^9^/L)	5.81 (4.91,6.83)	6.34 (5.41,7.40)	−13.730	<0.001	6.10 (5.20,7.17)	6.69 (5.77,7.72)	−14.980	<0.001
SBP (mmHg)	120 (110,132)	136 (122,151)	−28.521	<0.001	128 (117,142)	134 (123,147)	−12.480	<0.001
DBP (mmHg)	71 (64,78)	77 (70,86)	−21.709	<0.001	76 (69,84)	81 (74,90)	−16.262	<0.001
TG (mmol/L)	0.83 (0.60,1.18)	1.52 (1.07,2.11)	−40.024	<0.001	1.03 (0.75,1.47)	1.73 (1.20,2.53)	−34.564	<0.001
TC (mmol/L)	4.69 (4.15,5.34)	5.21 (4.60,5.88)	−19.221	<0.001	4.61 (4.09,5.21)	4.87 (4.33,5.48)	−11.856	<0.001
HDL-C (mmol/L)	1.54 (1.33,1.78)	1.28 (1.12,1.49)	−28.916	<0.001	1.27 (1.09,1.48)	1.09 (0.94,1.26)	−25.385	<0.001
LDL-C (mmol/L)	2.80 (2.32,3.37)	3.36 (2.83,3.96)	−24.385	<0.001	2.92 (2.46,3.44)	3.18 (2.68,3.70)	−12.513	<0.001
FPG (mmol/L)	4.97 (4.71,5.26)	5.37 (5.03,5.90)	−30.224	<0.001	5.16 (4.85,5.51)	5.39 (5.03,5.97)	−17.626	<0.001
ALT (U/L)	13 (10,17)	19 (14,26)	−33.115	<0.001	17 (13,23)	25 (18,36)	−29.760	<0.001
AST (U/L)	17 (15,20)	20 (17,24)	−19.249	<0.001	19 (16,22)	21 (18,25)	−14.857	<0.001
GGT (U/L)	14 (11,18)	21 (16,29)	−35.491	<0.001	21 (16,30)	33 (24,49)	−30.714	<0.001
BUN (mmol/L)	4.50 (3.76,5.33)	4.82 (4.09,5.71)	−10.698	<0.001	5.23 (4.46,6.13)	5.21 (4.47,6.08)	− 1.083	0.279
Scr (μmol/L)	53 (48,58)	53 (48,59)	−1.531	0.126	73 (67,80)	73 (66,80)	−1.549	0.121
UA (μmol/L)	253 (221,289)	297 (258,343)	−28.558	<0.001	343 (299,390)	383 (334,438)	−20.998	<0.001

Data were expressed as median (interquartile range) and numbers (percentage) as appropriate.

*Abbreviations*: *NAFLD* nonalcoholic fatty liver disease, *BMI* body mass index, *WC* waist circumference, *WHtR* waist-to-height, *LAP* lipid accumulation product, *CMI* cardiometabolic index, *WBC* white blood cell count, *SBP* systolic blood pressure, *DBP* diastolic blood pressure, *TG* triglyceride, *TC* total cholesterol, *HDL-C* high density lipoprotein cholesterol, *LDL-C* low density lipoprotein cholesterol, *FPG* fasting blood glucose, *ALT* alanine aminotransferase, *AST* aspartate aminotransferase, *GGT* γ-glutamyltransferase, *BUN* blood urea nitrogen, *Scr* serum creatinine, *UA* uric acid

^a.^ Comparisons of continuous variables between groups were tested by Mann-Whitney U test due to skewed distribution and categorical variables between groups were tested by chi-squared test

### Relationship of LAP and CMI with risk of NAFLD

**Fig. 1:**
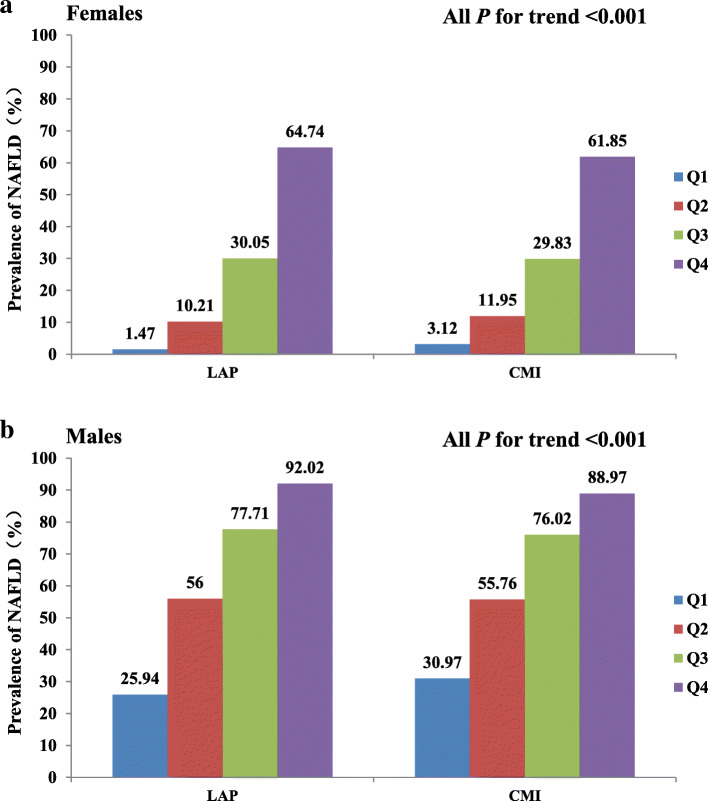
Prevalence of NAFLD according to the quartiles of LAP and CMI. Abbreviations: NAFLD, nonalcoholic fatty liver disease; LAP: lipid accumulation product; CMI, cardiometabolic index

Based on the sex-specific quartile analysis, a dose-response association existed between LAP and CMI with NAFLD risk (Fig.1). Regardless of sex, NAFLD prevalence increased progressively with ascending quartile of LAP and CMI (all *p* for trend <0.001). In the case of females, NAFLD prevalence was 44.04 and 19.82-folds higher across LAP and CMI quartiles, respectively. In males, NAFLD prevalence was 3.55 and 2.87-folds higher across LAP and CMI quartiles, respectively.

### Multivariate logistic regression assessing LAP and CMI influence on NAFLD identification

In multivariate logistic regression analyses, LAP and CMI expressed as either continuous variables or quartiles, markedly associated with NAFLD in all models (Table 2).

**Table 2:** Multivariate logistic regression of LAP and CMI for NAFLD

Variables	Odds Ratio (95%CI)					
	Crude	*P* value	Model 1	*P* value	Model 2	*P* value
Females						
LAP level (per 1 SD change)	1.068 (1.065,1.072)	<0.001	1.035 (1.031,1.039)	<0.001	1.026 (1.022,1.030)	0.002
Quartiles of LAP						
Q1 (≤7.52)	1.000 (reference)		1.000 (reference)		1.000 (reference)	
Q2 (7.52–15.12)	7.639 (5.113,11.412)	<0.001	4.275 (2.831,6.456)	<0.001	3.437 (2.261,5.225)	<0.001
Q3 (15.12–29.60)	28.840 (19.609,42.417)	<0.001	9.736 (6.481,14.623)	<0.001	6.575 (4.327,9.991)	<0.001
Q4 (>29.60)	123.295 (83.913,181.159)	<0.001	24.822 (16.335,37.720)	<0.001	13.183 (8.512,20.417)	<0.001
*P* value for trend	<0.001		<0.001		<0.001	
CMI level (per 1 SD change)	23.521 (19.410,28.503)	<0.001	8.114 (6.642,9.913)	<0.001	3.110 (2.579,3.750)	<0.001
Quartiles of CMI						
Q1 (≤0.18)	1.000 (reference)		1.000 (reference)		1.000 (reference)	
Q2 (0.18–0.30)	4.211 (3.151,5.628)	<0.001	2.841 (2.100,3.843)	<0.001	2.246 (1.637,3.082)	<0.001
Q3 (0.30–0.53)	10.103 (17.371,13.247)	<0.001	6.338 (4.758,8.443)	<0.001	3.983 (2.946,5.387)	<0.001
Q4 (>0.53)	38.459 (50.347,65.909)	<0.001	18.746 (14.059,24.996)	<0.001	8.662 (6.371,11.778)	<0.001
*P* value for trend	<0.001		<0.001		<0.001	
Males						
LAP level (per 1 SD change)	1.063 (1.059,1.067)	<0.001	1.042 (1.038,1.046)	<0.001	1.033 (1.029,1.037)	<0.001
Quartiles of LAP						
Q1 (≤16.02)	1.000 (reference)		1.000 (reference)		1.000 (reference)	
Q2 (16.02–28.52)	3.632 (3.142,4.199)	<0.001	2.288 (1.925,2.721)	<0.001	1.790 (1.491,2.148)	<0.001
Q3 (28.52–50.00)	9.953 (8.501,11.653)	<0.001	5.087 (4.191,6.175)	<0.001	3.462 (2.810,4.265)	<0.001
Q4 (>50.00)	32.922 (26.775,40.481)	<0.001	13.048 (10.205,16.683)	<0.001	7.544 (5.748,9.902)	<0.001
*P* value for trend	<0.001		<0.001		<0.001	
CMI level (per 1 SD change)	9.435 (8.069,11.033)	<0.001	6.078 (5.162,7.156)	<0.001	3.069 (2.603,3.618)	<0.001
Quartiles of CMI						
Q1 (≤0.36)	1.000 (reference)		1.000 (reference)		1.000 (reference)	
Q2 (0.36–0.62)	2.810 (2.442,3.233)	<0.001	2.467 (2.100,2.899)	<0.001	1.779 (1.492,2.122)	<0.001
Q3 (0.62–1.05)	7.068 (6.068,8.234)	<0.001	5.247 (4.408,6.245)	<0.001	2.884 (2.379,3.496)	<0.001
Q4 (>1.05)	17.978 (14.967,21.595)	<0.001	12.278 (9.990,15.089)	<0.001	5.400 (4.297,6.786)	<0.001
*P* value for trend	<0.001		<0.001		<0.001	

*Abbreviations*: *NAFL*D nonalcoholic fatty liver disease, *LAP* lipid accumulation product, *CMI* cardiometabolic index. Crude: no adjustment; Model 1: adjusted for age, current smoking, regularly exercising, BMI. Model 2: adjusted for all the factors in Model 1 and WBC, SBP, TC, LDL-C, FPG, ALT, GGT, UA among males, additionally plus BUN among females

Among women, the ORs for 1 SD elevation of LAP and CMI were 1.068 (95%CI = 1.065–1.072) and 23.521 (95%CI = 19.410–28.503), respectively. Upon adjusting for several possible confounders (Model 2), it was still significant [LAP, OR = 1.026 (95%CI = 1.022–1.030) per 1 SD increment; CMI, OR = 3.110 (95%CI = 2.579–3.750) per 1 SD increment; all *P*<0.001]. For males, the ORs for 1 SD elevation in LAP and CMI were 1.063 (95%CI = 1.059–1.067) and 9.435 (95%CI = 8.069–11.033), respectively. Upon adjusting for several possible confounders (Model 2), it was still significant [LAP, OR = 1.033 (95%CI = 1.029–1.037) per 1 SD increment; CMI, OR = 3.069 (95%CI = 2.603–3.618) per 1 SD increment; all *P*<0.001].

Upon dividing LAP and CMI into quartiles, their relationship with NAFLD remained statistically significant. After adjusting for several possible confounders (Model 2), subjects in the largest LAP quartile displayed a 13.183-fold (95%CI = 8.512–20.417) NAFLD risk in females and 7.544-fold (95%CI = 5.748–9.902) NAFLD risk in males. Subjects in the largest CMI quartile displayed an 8.662-fold (95%CI = 6.371–11.778) NAFLD risk in females and 5.400-fold (95%CI = 4.297–6.786) NAFLD risk in males. All *P* values for this trend were less than 0.001 in both sexes.

### Diagnostic capacity of anthropometric indices for predicting NAFLD

Table 3 summarized the AUCs of various adiposity NAFLD markers by sex. In females, LAP displayed the largest AUC (0.860, 95%CI = 0.852–0.867) followed by CMI (0.833, 95%CI = 0.825–0.842), WHtR (0.826, 95%CI = 0.817–0.834), BMI (0.820, 95%CI = 0.814–0.825) and WC (0.819, 95%CI = 0.810–0.827). In males, LAP displayed the largest AUC (0.816, 95%CI = 0.806–0.825), followed by CMI (0.779, 95%CI = 0.769–0.789), BMI (0.777, 95%CI = 0.771–0.783), WC (0.772, 95%CI = 0.762–0.782) and WHtR (0.764, 95%CI = 0.753–0.774). Notably, in both sexes, LAP and CMI were more accurate than other obesity-related indexes in discriminating the presence of NAFLD.

**Table 3:** AUCs of various indexes for discriminating NAFLD by sex

Variables	AUC (95%*CI*)	*P* value	Cut-off value	Sensitivity (%)	Specificity (%)
Females					
LAP (cm.mmol/L)	0.860 (0.852,0.867)	<0.001	19.2	82.32	74.43
CMI	0.833 (0.825,0.842)	<0.001	0.34	81.92	68.69
WHtR	0.826 (0.817,0.834)	<0.001	0.47	80.54	69.57
BMI (Kg/m^2^)	0.820 (0.814,0.825)	<0.001	23.94	78.19	70.38
WC (cm)	0.819 (0.810,0.827)	<0.001	75	78.28	70.11
Males					
LAP (cm.mmol/L)	0.816 (0.806, 0.825)	<0.001	27.86	68.82	78.72
CMI	0.779 (0.769, 0.789)	<0.001	0.56	69.97	72.39
WHtR	0.764 (0.753, 0.774)	<0.001	0.48	74.38	65.91
BMI (Kg/m^2^)	0.777 (0.771,0.783)	<0.001	25.08	75.36	68.23
WC (cm)	0.772 (0.762, 0.782)	<0.001	83	73.99	66.79

Abbreviations: *AUC* area under the ROC curve, *95%* *CI* 95% confidence interval, *LAP* lipid accumulation product, *BMI* body mass index, *CMI* cardiometabolic index, *WC* waist circumference

## Discussion

Among the populous Chinese study population, the findings were as follows. First, the role of LAP was confirmed in the identification of NAFLD, after adjusting for major confounders in both sexes. Second, for the first time, CMI was found to be significantly associated with NAFLD prevalence, after adjusting for major confounders in both sexes. Thus, CMI appears to be a simple and promising tool in the early monitoring and targeted intervention of NAFLD. Third, both LAP and CMI exhibited a stronger correlation with NAFLD in females than in males. The tends of NAFLD risk to increase in females, particularly around the age of 55 years old, at which point most women underwent menopause. Post menopause, estrogen levels plummet and body fat distribution shifts to the abdominal region [9]. Estrogen is known to modulate lipid metabolism while suppressing vascular, inflammation, cell growth, and plaque advancement in premenopausal women. Menopausal initiates a cascade of biological and physiological alterations, which includes fat redistribution (i.e., accumulation of visceral fat), dyslipidemia and glucose intolerance, which are strongly correlated with enhanced IR, cardiovascular disease and NAFLD [20].

LAP, established by the National Nutrition Survey, is well accepted as a novel obesity-related index [4]. Compared to traditional obesity-related indexes, LAP comprehensively evaluates excessive lipid accumulation. In addition, LAP performed better than BMI in predicting the diabetes risk [21], and was a reliable predictor of insulin resistance. LAP also had a higher diagnostic accuracy in terms of MS, compared to BMI, WC and WHtR [22, 23]. In a cross-sectional study involving 40,459 subjects from southern China, LAP was shown to strongly associate with the diagnosis and severity of NAFLD [24], which was similar to this current findings from northern China. However, the best cut-off values for LAP in predicting NAFLD were a bit different between northern and southern Chinese population. This might be due to potential heterogeneity in geographical environment, regional climate, dietary and living habits, and prevalence of overweight and obese population between these two regions.

NAFLD pathogenesis may be attributed to abdominal obesity and high TG levels. In patients with abdominal obesity, visceral adipocytes induce synthesis of a variety of cytokines like interleukim-6 and tumor necrosis factor-α, which promote macrophage infiltration and chronic inflammation [25]. Simultaneously, adipocytes secrete adipose factor chemokine, which regulates carbohydrate and lipid metabolism [26]. Chronic inflammation can affect the signal transduction pathway of surrounding cells such as T cells (including invariant natural killer cells), eosinophils, B-regulatory cells (Bregs)12 and macrophages, leading to insulin resistance, liver steatosis, and eventually promotes NAFLD development [27]. In addition, omega-3 fatty acids, usage can usually lower liver steatosis by reducing TG levels, thus supporting the role of elevated TG levels in the development of NAFLD [28].

CMI is a recently developed index, based on TG/HDL-C and WHtR values that could easily be achieved during health check-ups [10]. As previously mentioned, multiple studies suggested that CMI was strongly associated with obesity-related metabolic diseases, such as diabetes and cardiovascular disease [12–15]. All components of CMI are also considered in the criteria for MS, including abdominal obesity and dyslipidemia. WHtR is an abdominal obesity measurement index that is strongly associated with lipid content and lipid distribution, and is superior to WC and BMI in the assessment of NAFLD [29]. Additionally, previous studies confirmed that TG/HDL-C was closely related to insulin resistance (IR), obesity and metabolic disorders and had a good predictive value for NAFLD diagnosis [30–32]. In this study, it was found that a larger CMI quartile was markedly and independently correlated with an enhanced NAFLD risk, based on a graded mode regardless of sex. In addition, the ROC analyses showed that CMI presented an adequate diagnostic performance.

In a relatively recent study, NAFLD was shown to be mutual and bi-directional related to MS [33]. Multiple essential metabolic indicators that make up the CMI also participate in regulating fatty liver disease. Moreover, the theory of IR is the core of NAFLD pathogenesis [34, 35]. The relationship between CMI and NAFLD is somewhat unclear. Based on the present findings, IR may mediate the connection [36]. Prior studies confirmed that the abdominal fat and TG/HDL-C ratio were closely related to IR. Patients with abdominal obesity exhibit high levels of glucose and lipid oxidation, and releases free fatty acid (FFA). Once FFA exceeds the buffer capacity of the peripheral fat storage library, liver fat accumulation can accelerate development of IR and NAFLD [37, 38]. Other studies revealed that IR promoted secretion of very low-lipoprotein (VLDL) and TG, and reduced HDL-C levels [39, 40]. In addition, some researchers reported that IR can promote NAFLD development by inducing TG decomposition of within adipose tissue and simultaneously enhancing TG synthesis in the liver [41, 42]. On contrary, IR emergence accelerates sugar decomposition, leading to an enhancement in blood glucose and VLDL, which then promotes the release of excessive TC into the blood, thus raising serum TC levels [43, 44].

### Study strength and limitations

In this study, it was confirmed the role of LAP in identifying NAFLD, after adjusting for major confounders in both sexes. In addition, it was the first time to find that CMI was significantly associated with NAFLD prevalence, after adjusting for major confounders in both sexes. Third, both LAP and CMI exhibited a stronger correlation with NAFLD in females than in males. There were also some limitations in this study that deserved mention. First, the cross-sectional nature of this study only provided the correlation between LAP, CMI and NAFLD, but the cause of this association needs exploration via longitudinal investigations. Second, the patients were selected from the health check-up populations of Chinese adults. Thus, the conclusions might not be appropriate to people of other ethnicities or races. Thirdly, NAFLD was diagnosed via ultrasonography, which has limited sensitivity and unreliable detection of <5% liver fat infiltration [45]. Fourth, this study did not include subgroup analysis involving female menopausal status. Finally, there was no detailed division of various degrees of fatty liver, so it was impossible to evaluate the diagnostic value of CMI in nonalcoholic steatohepatitis and liver fibrosis. At present, liver biopsy, being an invasive test, is not typically required for NAFLD diagnosis [46]. Instead, scientists designed noninvasive approaches like computed tomography and magnetic resonance imaging to detect NAFLD. However, these approaches are both costly and time-consuming, and are not suitable for the screening and application of large-scale population [47]. Thus, simple and convenient new synthetic biological indexes, namely LAP and CMI, are of great significance to the general screening of NAFLD.

## Conclusions

In conclusion, LAP and CMI positively and independently correlated with NAFLD risk, and had a stronger correlation in women. The novel and clinically effective markers LAP and CMI offer simple and easy approach to the early identification of people at an elevated risk of NAFLD. The conclusions emphasized the significance of personalized treatment plans, using early detection markers LAP and CMI and their sex-specific qualities in preventing NAFLD.

## Data Availability

The datasets used and/or analyzed during the current study are available from the corresponding author on reasonable request.

## Abbreviations

NAFLD: nonalcoholic fatty liver disease
BMI: body mass index
WC: waist circumference
WHtR: waist-to-height
LAP: lipid accumulation product
CMI: cardiometabolic index
WBC: white blood cell count
SBP: systolic blood pressure
DBP: diastolic blood pressure
TG: triglyceride
TC: total cholesterol
HDL-C: high density lipoprotein cholesterol
LDL-C: low density lipoprotein cholesterol
FPG: fasting blood glucose
ALT: alanine aminotransferase
AST: aspartate aminotransferase
GGT: γ-glutamyltransferase
BUN: blood urea nitrogen
Scr: serum creatinine
UA: uric acid
